# KSHV Genome Replication and Maintenance

**DOI:** 10.3389/fmicb.2016.00054

**Published:** 2016-02-01

**Authors:** Pravinkumar Purushothaman, Prerna Dabral, Namrata Gupta, Roni Sarkar, Subhash C. Verma

**Affiliations:** Department of Microbiology and Immunology, School of Medicine, University of Nevada, RenoReno, NV, USA

**Keywords:** KSHV, LANA, latency, LBS, replication, pre-RC, ori-P, ori-A

## Abstract

Kaposi's sarcoma associated herpesvirus (KSHV) or human herpesvirus 8 (HHV8) is a major etiological agent for multiple severe malignancies in immune-compromised patients. KSHV establishes lifetime persistence in the infected individuals and displays two distinct life cycles, generally a prolonged passive latent, and a short productive or lytic cycle. During latent phase, the viral episome is tethered to the host chromosome and replicates once during every cell division. Latency-associated nuclear antigen (LANA) is a predominant multifunctional nuclear protein expressed during latency, which plays a central role in episome tethering, replication and perpetual segregation of the episomes during cell division. LANA binds cooperatively to LANA binding sites (LBS) within the terminal repeat (TR) region of the viral episome as well as to the cellular nucleosomal proteins to tether viral episome to the host chromosome. LANA has been shown to modulate multiple cellular signaling pathways and recruits various cellular proteins such as chromatin modifying enzymes, replication factors, transcription factors, and cellular mitotic framework to maintain a successful latent infection. Although, many other regions within the KSHV genome can initiate replication, KSHV TR is important for latent DNA replication and possible segregation of the replicated episomes. Binding of LANA to LBS favors the recruitment of various replication factors to initiate LANA dependent DNA replication. In this review, we discuss the molecular mechanisms relevant to KSHV genome replication, segregation, and maintenance of latency.

## Introduction

Kaposi's sarcoma-associated herpesvirus (KSHV), also known as human herpesvirus 8 (HHV-8), belongs to the γ-herpesvirus family. It is one of the seven recognized human cancer causing viruses (Moore and Chang, 2010). KSHV is etiologically associated with Kaposi's sarcoma (KS) as well as two other cell proliferative cancers, Primary Effusion Lymphoma (PEL) and Multicentric Castleman's Disease (MCDs) (Chang et al., 1994; Cesarman et al., 1995). KS lesions are highly complex and are characterized by proliferating spindle-shaped endothelial cells (ECs), neovascular structures, leukocyte infiltrations (monocytes, lymphocytes, and mast cells), and an abundance of inflammatory cytokines (ICs), growth factors, angiogenic factors, and invasive factors (Chang et al., 1994; Valiya Veettil et al., 2014). KSHV genome exists as a linear dsDNA in the virion particles and its size ranges from 165 to 170-kb (Renne et al., 1996; Neipel et al., 1998). The large KSHV genome consists of a ~137 kb long unique region (LUR), encompassing the KSHV ORFs, flanked by a 30-kb terminal repeat sequences, which are reiterated copies of 801 bp sequence with high GC-content, important for multiple functions of the viral life cycle (Renne et al., 1996; Russo et al., 1996; Duprez et al., 2007). Once transported to the nucleus, the linear viral DNA rapidly circularizes and is maintained as an episome upon infection.

## KSHV life cycle

**Figure 1:**
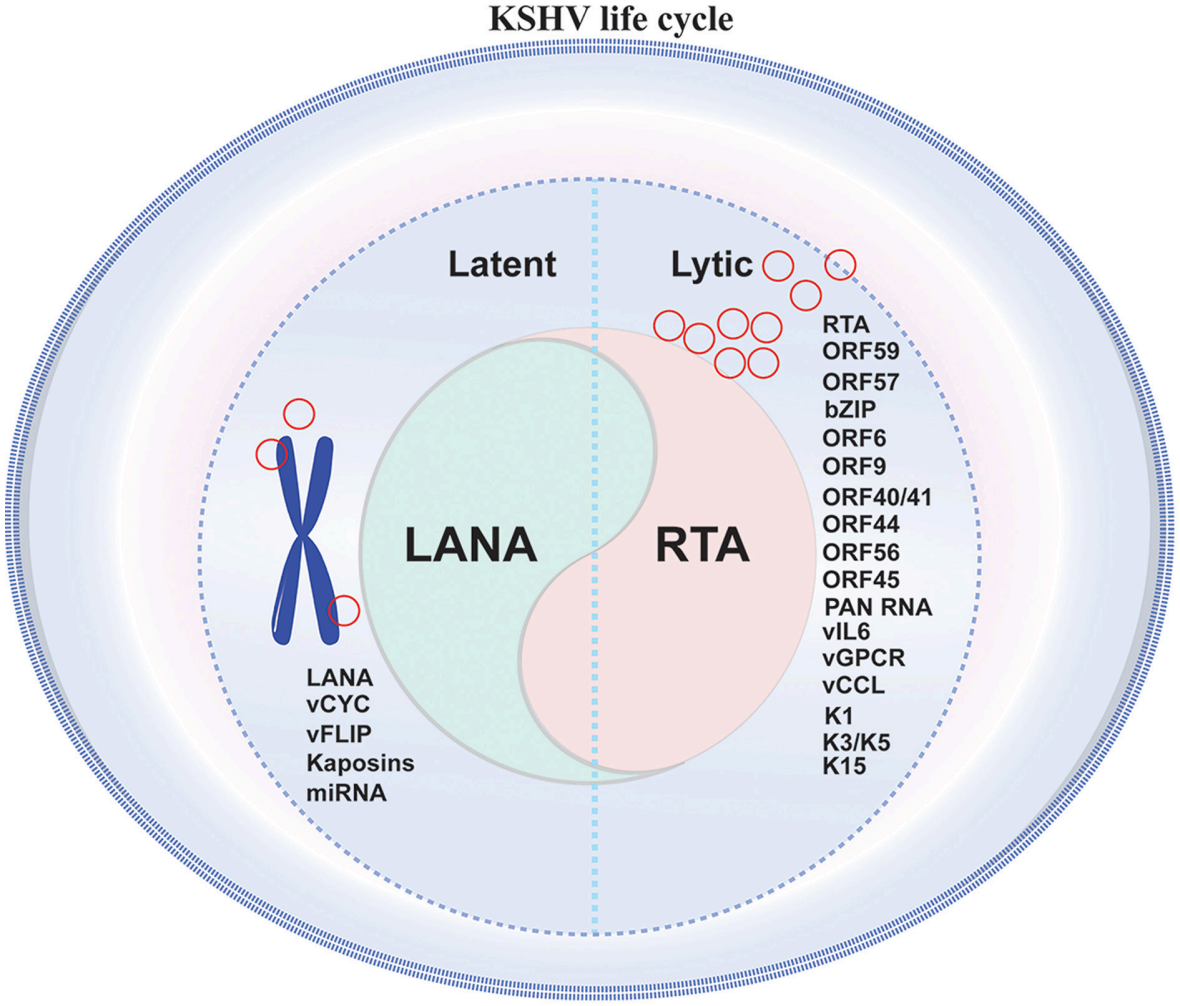
**Schematic representation of KSHV life cycle in infected cell**. KSHV life cycle comprises of two distinct phases of infection namely a short lytic phase and a predominant latent phase. Maintaining a perfect balance between latent and lytic phase is important for the long-term persistence of the virus in the host, additionally specific gene products from both the expression programs play crucial roles in the pathogenesis of KSHV-associated disease.

Like many other members of the γ-herpesvirus family, KSHV life cycle is divided into two distinct phases of infection, the lytic phase and the default latent phase, which are characterized by the patterns of viral gene expression (Ye et al., 2011). Both latent and lytic cycle replication are important for the long-term persistence of the virus in the host, and the gene products from both expression programs serve critical roles in the pathogenesis of KSHV-associated disease (Cesarman, 2002; Figure 1).

During latency, KSHV expresses a limited number of viral genes, such as ORF73 (latency-associated nuclear antigen 1 [LANA-1]), ORF72 (viral Cyclin [vCyclin]), ORF71 (K13/vFLIP), and ORFK12 (kaposins A, B, and C), along with 12 distinct microRNAs, to facilitate the establishment of lifelong latency in its host and survival against the host innate, and adaptive immune surveillance mechanisms (Staskus et al., 1997; Dittmer et al., 1998; McClure and Sullivan, 2008). KSHV encodes >86 open reading frames (ORFs), which includes at least 22 potential immunomodulators, K3, K5, K9, K11.1, and anti-apoptotic K7 (viral Bcl-2) (Russo et al., 1996; Neipel et al., 1997), which regulate cytokine secretion, antagonize host interferon (IFN)-mediated antiviral responses, and regulate immune evasion (Jenner et al., 2001; Valiya Veettil et al., 2014). During latency, KSHV genome persists as a circular episome that subsequently associates with cellular histones and exists as a non-integrated minichromosome in the nucleus of infected cells (Toth et al., 2013) with the expression of limited viral genes to maintain the genome in dividing cells and to limit host immune responses while enhancing cell survival and virus persistence (Cai et al., 2010b). The latency associated viral products have an essential role in the development of KSHV-associated malignancies because most tumor cells in KS, PEL, and MCD are latently infected by KSHV (Cesarman, 2002; Cotter and Robertson, 2002). KSHV latent cells can be reactivated into lytic replication by specific intracellular or extracellular stimuli, chemicals that affect chromatin regulatory factors such as valproic acid, hypoxia, 12-O-tetradecanoylphorbol-13-ace-tate (TPA), and butyrate (Yu et al., 1999; Shaw et al., 2000; Davis et al., 2001), histone deacetylases (HDACs), DNA methyltransferases (DNMTs), and histone acetyltransferases (HATs) (Chen et al., 2001; Lu et al., 2003; Xie et al., 2008), as a result of which, the viral episome gradually relaxes its compact chromatin structure, leading to the expression of all viral genes and the production of infectious virions particles (Iyengar and Farnham, 2011). This indicates that transition of KSHV life cycle involves viral chromatin remodeling from the heterochromatin to the euchromatin state (Zhang et al., 2014).

The first lytic genes to be expressed during lytic cycle replication in B lymphocytes is an immediate early (IE) gene, RTA (ORF50) (Gradoville et al., 2000), which triggers the expression of early genes required for viral DNA replication followed by the expression of late genes (Jenner et al., 2001; Rossetto et al., 2007; Verma et al., 2015). Sequential expression of these viral lytic products promotes cell proliferation, angiogenesis, and local inflammation due to virus production and shutdown of host cell protein synthesis, leading to the initiation and progression of KS tumors (Pan et al., 2004; Sadagopan et al., 2007; Ye et al., 2007). In latently infected cells, RTA (replication and transcription activator) expression is tightly restricted, suggesting that transcriptional repression of RTA gene is needed to establish latency in the host (Katano et al., 2001). Chromatin modification and nucleosome positioning seem to be essential for inactivation as well as activation of latent genes (Lu et al., 2006; Pantry and Medveczky, 2009). Recent genome-wide analysis using ChIP-on-chip in conjunction with immunoprecipitation of methylated DNA examining the epigenetic landscape of KSHV genomes, showed that the KSHV genomes undergo methylation at CpG nucleotide accompanied by specific histone modification marks leading to a rapid establishment of latency and silencing of lytic gene expression (Günther and Grundhoff, 2010; Toth et al., 2010, 2013).

## KSHV latency program

Modulation by viral factors plays a key role in maintaining viral latency. Latent transcriptional program that has largely been carried out in more than 90% of infected cells, has led to the characterization of a major latency locus that is abundantly and consistently transcribed in all latently KSHV-infected cells (Dittmer et al., 1998; Burýsek and Pitha, 2001). Since LANA, vCyclin, vFLIP, and the miRs are located in the latency locus of KSHV genome and are expressed during latency, they are likely to contribute to KSHV latency (Burýsek and Pitha, 2001; Dittmer et al., 1998). They are also involved in KSHV induced malignant transformation and oncogenesis by promoting cell growth and survival and the induction of inflammatory cytokines.

### Latency-associated nuclear antigen (LANA)

Latency-associated nuclear antigen (LANA) encoded by ORF73 is the major latent protein strongly expressed in all forms of KSHV-associated malignancies throughout the viral life cycle (Rainbow et al., 1997; Gao et al., 1999). LANA is essential for KSHV episomal replication, maintenance, and efficient segregation of episomal DNA into the daughter cells during mitosis (Hu et al., 2002; Si et al., 2008). LANA is a multifunctional nuclear protein with 1162 amino acids and 220–230 kDa in size that interacts with various cellular as well as viral proteins, including tumor suppressors, p53 (Friborg et al., 1999) and pRb (Radkov et al., 2000), transcription factors such as ATF4/CREB2 (Lim et al., 2000) and STAT3 (Muromoto et al., 2006), cellular signal transducer, GSK-3ß (Fujimuro and Hayward, 2003; Fujimuro et al., 2003), chromatin-binding proteins such as HP1 (Lim et al., 2003), histone H2A/B (Barbera et al., 2006), MeCP2 (Matsumura et al., 2010), and Brd4 (Ottinger et al., 2006).

### LANA mediated cellular signaling

**Figure 2:**
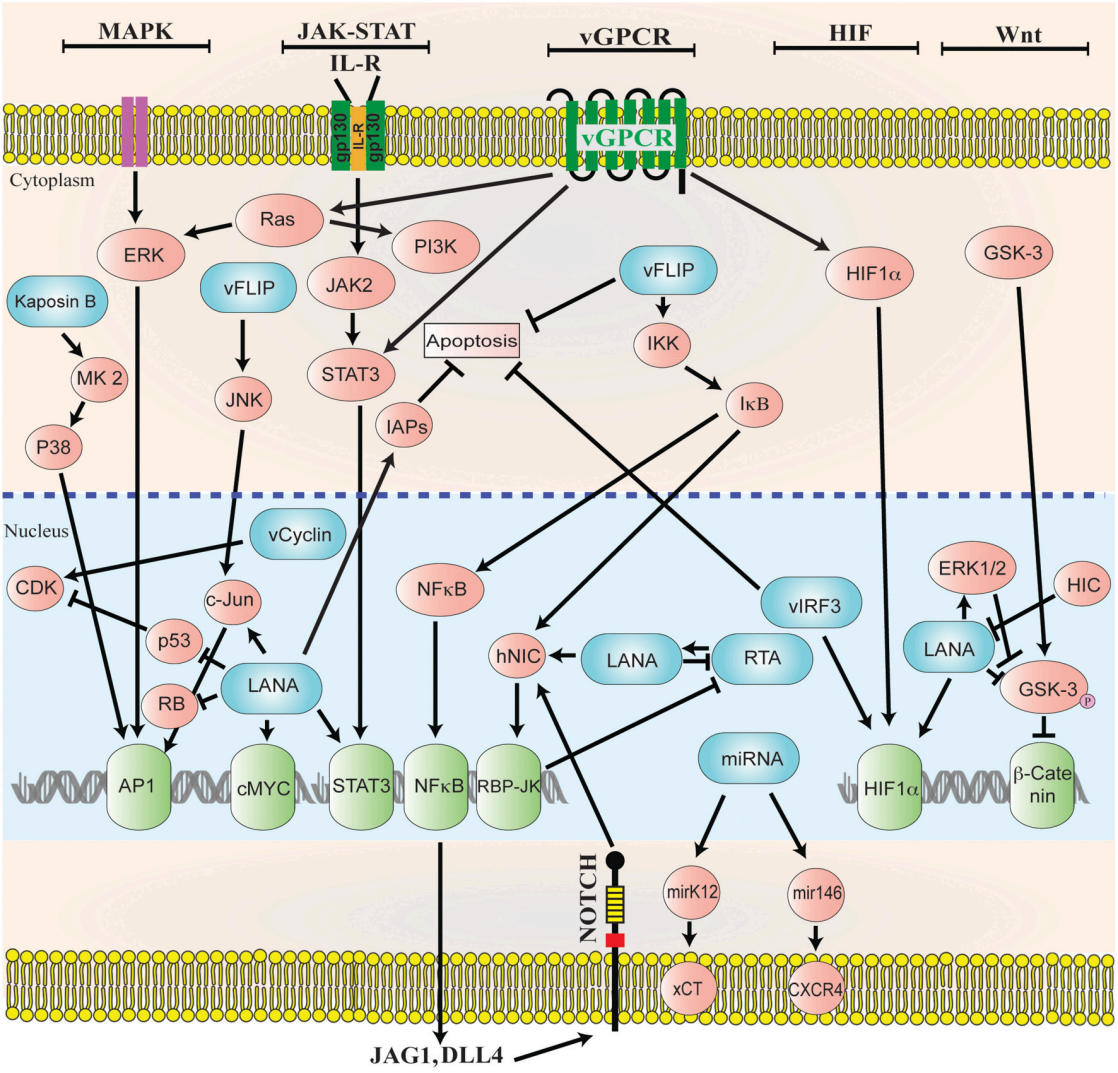
**Diagrammatic representation of the cellular signaling pathways in maintaining latency**. KSHV genome persists as a latent episome within the infected cells by expressing a limited number of viral genes during latency. For a successful establishment of latency, KSHV manipulates and deregulates multiple viral and cellular signaling pathways. KSHV latent genes, including LANA, vFLIP, miRNA, and vCyclin activate and maintain various cytokine-mediated cell proliferation and tumorigenesis pathways, such as MAPK, JAK/STAT, MEK/ERK, PI3K/AKT/mTOR, Notch, Wnt, cMyc, p53, RB, and NF-κB, to maintain latent infection.

For the successful establishment of latency, KSHV is armed to manipulate multiple viral and cellular signaling pathways to repress KSHV reactivation and to escape the host immune surveillance (reviewed in, Verma et al., 2007c; Cai et al., 2010b). KSHV LANA directly deregulates various cellular signaling pathways (Verma et al., 2004; Lan et al., 2005) such as *MAPK, JAK/STAT, MEK/ERK, PI3K/AKT, Notch*, and Wnt signaling to establish latency (Figure 2).

### MAPK pathway

The majority of the pathways activated after KSHV infections include, JNK, MEK-ERK, and p38 mitogen activated protein kinase (MAPK). The establishment of persistent infection depends on the activation of these cellular signaling pathways (Sharma-Walia et al., 2005; Pan et al., 2006). Similarly, reactivation of latently infected cells leads to the activation of MAPK pathways (Ford et al., 2006; Yu et al., 2007; Xie et al., 2008). KSHV LANA is involved in the activation of the serum response element and MAPK pathways through its interaction with a mediator complex (Roupelieva et al., 2010). During *de novo* KSHV infection heat shock protein 90 (hsp90) triggers latent gene expression and also acts as a cofactor for MAPK activation by localizing to the cellular surface (Qin et al., 2010a). Recently, a screening of the human kinome identified the significance of MSK1/2-CREB1 pathway in KSHV mediated lytic replication after the onset of infection (Cheng et al., 2015). These studies suggest that MAPK pathways play a critical role in KSHV infection as well as in lytic reactivation.

### JAK-STAT pathway

The cellular growth, differentiation and immune response is controlled by cytokine dependent JAK-STAT signaling. Constitutive activation of the receptor associated Janus tyrosine kinases are the consequence of KSHV infection leading to the phosphorylation of signal transducers and the activators of transcription (STATs) (Punjabi et al., 2007). The expression of gp130 receptor is induced during KSHV infection leading to the phosphorylation of JAK2/STAT3 (Punjabi et al., 2007; Morris et al., 2008). The role of LANA and vGPCR has been well documented in regulating JAK2/STAT3 signaling, which ultimately leads to the release of various angiogenic factors (Burger et al., 2005; Muromoto et al., 2006). The role of activated IL13/STAT6 signaling contributes to the cellular survival and proliferation after KSHV infection (Wang et al., 2015). The inhibition of this pathway leads to the inhibition of PELs survival and proliferation (Wang et al., 2015). KSHV LANA inhibits IL4-STAT6 signaling during apoptotic stress and in order to establish latency (Cai et al., 2010a). Also, IL-4/STAT6 signaling can regulate the reactivation of KSHV (Zeng et al., 2007).

### Notch signaling

Notch signaling pathway is involved in the determination of cell fate during stem cell maintenance, tissue homeostasis and development (Artavanis-Tsakonas et al., 1999; Lai, 2004). The latently infected cells are driven toward angiogenesis by LANA through targeting Hey1 (notch signaling effector; Wang et al., 2014). The levels of the components belonging to the Notch signaling cascades are higher in KS lesions and the experimental lesions are sensitive to the Notch pathway inhibitors (Curry et al., 2005, 2007). Notch signaling pathway is essential for KSHV's entry into lytic phase as well as for the cellular survival of the infected cells. The RTA protein of KSHV binds to RBP-Jκ, which is an essential component of Notch signal transduction pathway (Liang et al., 2002; Persson and Wilson, 2010). LANA targets sel10 protein in order to stabilize the activated forms of Notch receptors (Lan et al., 2007). KSHV infection increases the expression of Notch ligands (JAG1 and DLL4) through the expression of KSHV genes during latent and lytic phases of infection (Emuss et al., 2009). These studies conclusively show that KSHV infection influences cellular differentiation and proliferation by regulating Notch signaling cascade.

### HIF signal transduction

Hypoxia-inducible factor (HIF) is one of the transcriptional regulators, which leads to the transcription of various genes related to angiogenesis and tumorous growth. HIF is heterodimeric in structure with α (inducible) and β (constitutively expressed) subunits (Ke and Costa, 2006). Generally, the isoforms of HIFα has been categorized into three forms (HIF1α, HIF2α, and HIF3α) in humans (Ke and Costa, 2006). HIFα is hydroxylated and ubiquitinated in the presence of oxygen (Maxwell et al., 1999; Ravi et al., 2000), whereas the hydroxylation of HIFα is suppressed under hypoxic conditions leading to its enhanced stability and transcription of downstream genes involved in glycolysis, erythropoiesis and angiogenesis (Seagroves et al., 2001; Lee et al., 2004). The HIFα is highly expressed due to the induction of HIF signaling in various cancerous cells with vascularized and tumorous properties (Zhong et al., 1999; Zagzag et al., 2000). The expression of HIF1α and HIF2α are higher in latently infected cells during KSHV infection (Carroll et al., 2006; Cai et al., 2007). Also, the hypoxia mediated KSHV reactivation is induced through KAP1 inhibition. The suppression of KAP1 leads to the increased binding of RBP-Jκ with the HIF-1α complex thereby driving RTA expression (Zhang et al., 2014). KSHV encoded latent proteins; LANA and vIRF3 stabilize HIFα through protein-protein interaction (Cai et al., 2006; Shin et al., 2008). The stabilization of HIF1α through LANA has also been seen in the samples from the patients with KS (Long et al., 2009). Furthermore, VEGF expression has been reported to be upregulated through vGPCR via HIF1α induction during KSHV infection (Sodhi et al., 2000). Altogether, it is clear from the above studies that KSHV controls HIF1α expression for a successful establishment of infection.

### Wnt signaling pathway

The modulation of host immune system through KSHV includes the usage of cellular signaling components belonging to Wnt pathway. KSHV manipulates Wnt signaling cascade in order to drive the transcription of its own genes, which modulates downstream signaling after entry into the host cells. The involvement of LANA in the stabilization of β-catenin is conducted through its interaction with GSK-3β thereby trans-locating GSK-3β to the nucleus. This suppresses β-catenin phosphorylation in the cytoplasm and therefore leads to an increased TCF/LEF-dependent transcription (Fujimuro and Hayward, 2003, 2004). The involvement of KSHV encoded protein vGPCR in the activation of canonical Wnt/β-catenin signaling pathway has been reported in one of the recent studies (Angelova et al., 2014). The host cellular proteins I-mfa (inhibitor of MyoD family), I-mfa domain proteins and HIC (human I-mfa domain-containing protein) negatively regulate the activity of LANA in suppressing GSK-3β activity and also by inhibiting Wnt signaling activated by LANA (Kusano and Eizuru, 2010). Thus, KSHV mediated cellular transformation is controlled by LANA dependent β-catenin dysregulation.

### LANA mediated suppression of lytic reactivation

It has been proposed that LANA negatively regulates the transcription of viral lytic genes during the establishment of latency (Li et al., 2008). LANA inhibits RTA expression, one of the key viral lytic genes, by repressing the transcriptional activity of RTA promoter, where LANA associates with recombination signal sequence-binding protein Jκ (RBP-Jκ) and represses the RTA promoter through the RBP-Jκ binding site existing within its promoter (Lan et al., 2004, 2005; Lu et al., 2006). Another transcriptional repressor that controls chromosomal remodeling, Krüppel-associated box domain-associated protein 1 (KAP1), has been identified to play an important role in regulating KSHV latency by associating with the transcriptional promoters and repressing the lytic gene expression (Chang et al., 2009). In recent studies it has been proved that LANA can recruit KAP1 to the RTA promoter region of the KSHV genome to silence the expression of lytic genes to facilitate the establishment of KSHV latency (Sun et al., 2014a). Recent studies also suggested an involvement of LANA in the regulation of SUMOylation, its interaction with the protein related to post-translational modification of histones by the small ubiquitin-like modifier (SUMO), plays an important role in epigenetic control of gene transcription, DNA repair and replication (Cai et al., 2013). LANA contains a unique SUMO-interacting motif (LANA^SIM^) specific for its interaction with SUMO-2 and hence describes the first mechanism where the latent protein selectively recruits a cellular transcriptional complex for viral episome maintenance, modulation of viral chromatin leading to gene silencing during latent infection (Cai et al., 2013; Chang and Kung, 2014).

### vCyclin

vCyclin (vCYC) encoded by ORF72 is a viral homolog of cellular cyclin D, forms an active kinase complex with cellular cyclin-dependent kinase 6 (CDK6) and regulates cell cycle and cell proliferation by phosphorylating pRb protein, histones H1, CDK inhibitor (cdki), and p27 (Kip1) through vCyclin-CDK6 complex formation (Direkze and Laman, 2004; Van Dross et al., 2005). Thus, vCyclin might contribute to KSHV latency by promoting cellular proliferation. Studies show that vCyclin-CDK6 complex also mediates phosphorylation of nucleophosmin (NPM) to facilitate the interaction of NPM with LANA for the recruitment of HDAC1 to promote KSHV latency (Sarek et al., 2010).

### vFlip

vFLIP (viral Fas-associated protein with death domain-like interleukin-1β-converting enzyme/caspase-8-inhibitory protein), also known as K13, encoded by ORF71 interacts with several NF-κB-related signaling proteins and activates one of the key cellular survival pathways, the NF-κB pathway, in latently infected PEL cells to enhance cell proliferation and survival during latency by inhibiting viral lytic replication (Guasparri et al., 2004; Ye et al., 2008). This is achieved via binding to the inhibitor of kB-kinase γ (IKK γ) thus leading to an activation of the NF-κB pathway (Israël, 2010). It was also shown that KSHV mutant lacking the vFLIP gene inhibits the expression of RTA and suppress KSHV lytic cycle (Yang et al., 2011).

### K12/kaposins

Kaposins are another set of viral proteins expressed during latency, are comprised of Kaposin A, B, and C (Sadler et al., 1999). Amongst Kaposins, Kaposin B has been reported to promote tumor microenvironment by increasing cytokine expression; via it's binding with MK2 and activation of p38/MK2 pathway, resulting in the inhibition of cytokine mRNA degradation (McCormick and Ganem, 2005).

### Viral microRNAs (miRNAs)

These are single-stranded 20–23 nucleotide long noncoding RNA molecules involved in gene expression, which play critical role in the maintenance of KSHV latency by regulating the viral lytic genes and the host survival pathways (Boss et al., 2009; Lei et al., 2010a). The latency locus of KSHV genome encodes 12 pre-miRNAs, that further produce 18 mature miRNAs, and are highly conserved in all isolates of KSHV (for review, see Marshall et al., 2007; Uppal et al., 2014). All these KSHV miRNAs regulate several cellular factors such as NF-κB and IκBα, target RTA and inhibit the activation of viral lytic gene promoters and contribute toward the expression of latent genes (Feldman et al., 2014).

### miRNA mediated cellular signaling

KSHV encodes 12 pre-miRNAs generating 18 different highly conserved miRNAs (a single-nucleotide-edited miRNA, 16 different 5p or 3p miRNAs; Marshall et al., 2007; Lin et al., 2010). Maintenance of KSHV latency after infection requires the interaction of these virally encoded miRNA with various cellular factors. Among the viral miRNAs, miR-K12-11 suppresses BACH-1 by inducing xCT expression in the macrophages and endothelial cells infected with KSHV (Qin et al., 2010b) and miR-132 suppresses the expression of p300 (transcriptional coactivator) thereby regulates the innate antiviral immunity (Lagos et al., 2010). Also, the suppression of CXCR4 expression is mediated through miR-146a upregulation driven by viral FLICE inhibitory protein vFLIP (Punj et al., 2010). Apart from this KSHV miRNA suppresses the expression of leucine zipper transcription factor MAF (musculoaponeurotic fibrosarcoma oncogene homolog) after infection (Hansen et al., 2010). Also, the virally encoded miRNAs can suppress the protein expression of breakpoint cluster region by upregulating Rac1 activity. Additionally, Bcr knockdown in BCBL-1 cells (KSHV infected latent cells) showed an increase in RTA levels suggesting a contribution of KSHV miRNA in lytic reactivation (Ramalingam et al., 2015). The functional roles of KSHV miRNA (miR-K1) includes a downregulation of p21 expression thereby leading to the attenuation of cell cycle arrest generated through p21 (Gottwein and Cullen, 2010). miR-K1 is also involved in the regulation of both host and viral factors. It can regulate IκBα (NF-κB inhibitor) and the replication of virus by targeting the 3′UTR of its transcripts (Lei et al., 2010b). According to one of the studies, the cluster of KSHV miRNAs can suppress the transcription of ORF50 (RTA) (Lu et al., 2010). Based on these studies, it could be concluded that KSHV encoded miRNA plays a significant role in the establishment of persistent viral infection.

## Mechanism of KSHV latent DNA replication

In order to perpetuate itself in the host during latency, KSHV genome has to be duly replicated and accurately passed onto the daughter cells (Ballestas et al., 1999; Cotter and Robertson, 1999; Barbera et al., 2006). LANA is essential for genome maintenance as well as partitioning of the episomes into the dividing cells (Collins and Medveczky, 2002; Ye et al., 2004). LANA directly binds to the TR region through its carboxy-terminus and associates with components of the human chromatin at its amino-terminus (Cotter and Robertson, 1999; Ballestas and Kaye, 2001; Barbera et al., 2006). During latency KSHV effectively maintains 50–100 genome copies per infected cell through highly coordinated replication of the viral genome along with the cellular DNA (Ueda et al., 2006; Verma et al., 2007b; Ballestas and Kaye, 2011). LANA interacts with and recruits various cellular proteins, for genome replication and segregation (Ballestas et al., 1999; Barbera et al., 2006; Cotter and Robertson, 1999). KSHV genome assembles host cellular replication machinery at the TR and other parts of the viral genome in a cell cycle dependent manner (Verma et al., 2007b; Uppal et al., 2014).

### DNA replication licensing

Cellular replication is a complex multi-step process and is precisely controlled by a replication licensing system that regulates cells to replicate only once every cell cycle (Tsuyama et al., 2005). DNA replication licensing is controlled by sequential assembly of several pre-replication complex (pre-RC) proteins onto origin of replication (ori), including origin recognition complex (ORC), Cdc6, Cdt1, and mini-chromosomal maintenance (MCM) helicase MCM 2-7 (Tsuyama et al., 2005). The replication-licensing complex is initiated during G1 phase, by direct binding of origin recognition complex (ORC) to the ori site followed by Cdt1 (Nishitani and Lygerou, 2002). This complex then recruits ring-shaped Mcm2-7 hexamers to the double stranded DNA, followed by cyclin dependent kinase 2 (CDK2) and CDC7, thereby inducing a necessary change in conformation to generate a pair of bidirectional replication forks which in turn recruit the replication factors, leading to the formation of an active replication complex (Hodgson and Blow, 2002; Nishitani and Lygerou, 2002). Further, to restrict DNA replication once per cell cycle, pre-RC is disassembled from the origins after the replication is initiated (Tsuyama et al., 2005). In addition cellular Cyclins and Geminins also inactivate Cdt1 to prevent the reassembly of pre-RC to restrict re-replication in same cell cycle (May et al., 2005).

Generally in eukaryotic cells, DNA replication is initiated simultaneously at multiple replication origins on a chromosome to complete the process within a stipulated time (Huberman and Riggs, 1966; Wyrick et al., 2001). However various studies suggest that not all origins are activated simultaneously, the frequency and timing of DNA replication is tightly regulated by the extent of heterochromatin at different cell cycle stages (Dimitrova and Gilbert, 1999; Klochkov et al., 2009; Schwaiger et al., 2010). The replication timing of KSHV genome is not fully understood (Ohsaki and Ueda, 2012). Recent studies using single molecule analysis of replicated DNA (Adam et al., 2015), demonstrated that KSHV genome also can initiate replication at multiple points throughout its genome along with the TR region (Verma et al., 2011). Nonetheless, KSHV TR region is significant in latent DNA replication, since TR contains a well-defined origin of replication, ori-P (Hu and Renne, 2005; Verma et al., 2007b; Ohsaki and Ueda, 2012). It is suggested that LANA dependent latent DNA replication at ori-P is most probably initiated at the middle or late S phase of the cell cycle (reviewed in Ohsaki and Ueda, 2012).

### KSHV ori-P

KSHV ori-P consists of two LANA-binding sites (LBS) LBS-1/2 (Hu and Renne, 2005; Verma et al., 2007a) and a 32-bp GC-rich segment (32-GC) (reviewed in Ohsaki and Ueda, 2012). KSHV encoded LANA is indispensable during latency and plays a crucial role in KSHV latent DNA replication. LANA binds directly to LBS-1/2 and the ori-P, with varying degrees of affinity (Garber et al., 2001; Grundhoff and Ganem, 2003; Hu and Renne, 2005). In cell culture system, it has been shown that expression of KSHV LANA alone is capable of maintaining and replicating KSHV TR containing episomes and synthetic plasmids (Ballestas and Kaye, 2001; Hu et al., 2002; Grundhoff and Ganem, 2003). KSHV encoded LANA, comprises of at least four functional domains: an N-terminal proline-rich chromosome binding region (CBD); a long glutamic acid-rich internal repeat domain; a putative leucine zipper region and a carboxy-terminal, TR binding domain (DBD) (Verma et al., 2007c; Ponnusamy et al., 2015). The C-terminal DBD binding region is capable of both dimerization and subsequent binding to specific LANA binding sequences (LBS-1, -2, and 32GC) to support ori-P replication (Hu et al., 2002; Ohsaki et al., 2009). The LBS sites within KSHV TR region are reiterating tandem sequences, therefore LANA needs to oligomerize for its cooperative association with LBS (Ballestas and Kaye, 2001; Komatsu et al., 2004; Domsic et al., 2013; Hellert et al., 2015). A recent study by Li et al showed that dorsal positively charged electrostatic patch within the LANA DBD plays a crucial role in KSHV latent DNA replication and genome maintenance (Han et al., 2010). LANA DBD interacts with BET (Bromodomain and Extra Terminal domain) family of proteins (Ottinger et al., 2006; You et al., 2006), comprising of BRD2, BRD3, and BRD4, which are known to associate with acetylated histones (Li et al., 2015). BET family of proteins are transcriptional regulators, which play an important role in cell cycle control. Through their N terminal Bromodomain, BRD2/RING3; directly associate with acetylated histone H4 on the chromosome. The extra-terminal domains provide additional docking station for several other proteins to bind to the chromatin (Pamblanco et al., 2001; Dey et al., 2003; Kanno et al., 2004; You et al., 2004). Brd2/RING3, regulates cell cycle through its interaction with E2F by stimulating the G1/S transition of the cell cycle (Denis et al., 2000). LANA is further shown to recruit BRD2/RING3 to chromatin through amino acids residues 1007 and 1055 (Platt et al., 1999; Mattsson et al., 2002; Ottinger et al., 2006). Furthermore, structural studies validated the functional significance of LANA DBD binding to LBS 1-2 (Hellert et al., 2015; Ponnusamy et al., 2015). Recent studies show that LANA DBD oligomerizes *in vitro* and binds to the LBS-1 and 2 and the binding induces a significant conformational change in the terminal repeat DNA (Ponnusamy et al., 2015). Apart from its association with LBS sequence, LANA interacts with various cellular factors and recruits host cellular machinery to effectively orchestrate the latent DNA replication (Si et al., 2006; Ballestas and Kaye, 2011).

### Viral DNA replication

**Figure 3:**
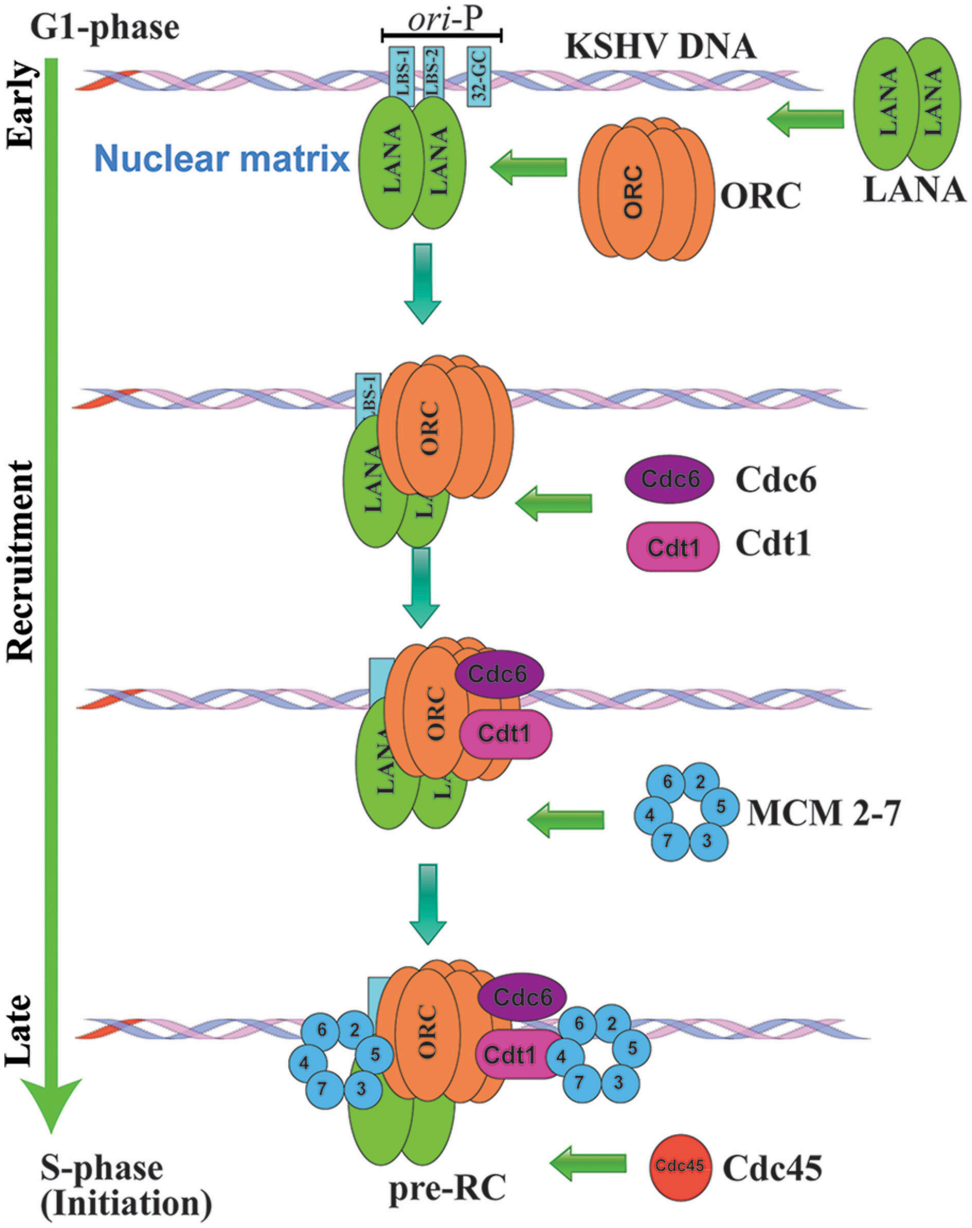
**Schematic representation of KSHV latent DNA replication initiation**. KSHV genomes replicate once per cell cycle during latent DNA replication and are perpetually segregated into the daughter cells. KSHV heavily depend on host cellular machinery for its latent DNA replication. Latent DNA replication is initiated by the direct association of LANA to LBS1/2 within the origin of latent DNA replication, ori-P, thereby triggering the recruitment various components of replication licensing and pre-replication complex (pre-RC) proteins to ori-P. The sequential assembly of pre-replication complex, including origin recognition complex (ORC), cell division cycle (Cdc6), Cdt1 and minichromosome maintenance proteins (MCM) within the nuclear matrix region initiate KSHV latent DNA replication.

KSHV replication is initiated by the direct association of LANA to the origin of latent DNA replication, ori-P, which in turn triggers the recruitment various components of the pre-replication complex (pre-RC) proteins to ori-P. The sequential assembly of pre-replication complex, including origin recognition complex (ORC), poly (ADP-ribose) polymerase 1 (PARP1), cell division cycle (Cdc6), and minichromosome maintenance proteins (MCM) within the nuclear matrix region, serves as the site of KSHV latent DNA replication in late G1 phase, (Figure 3; Lim et al., 2002; Ohsaki et al., 2004; Stedman et al., 2004; Verma et al., 2006).

Increasing evidences indicate that LANA DBD specifically recruits origin recognition complex (ORC2), Bromodomain Containing 2 (BRD2), H4-specific histone acetylase (HBO1), and CREB-binding protein (CBP) at the TR region (Stedman et al., 2004; Verma et al., 2006). Immediately after the recruitment of ORCs, DNA replication licensing factors including minichromosome maintenance proteins (MCM 2-7), which are putative helicases are assembled at the origin of replication (Stedman et al., 2004; Lei, 2005). LANA was found to be crucial for the recruitment of MCM3 to the TR, additionally siRNA mediated knock down of MCM3 expression showed significant reduction in TR-mediated replication (Stedman et al., 2004). Similarly, LANA was also shown to augment the loading of PCNA onto the DNA through its interaction with replication factor C (RFC) ATPase, thus contributing to the replication and persistence of the viral DNA (Sun et al., 2014b). PCNA is a homotrimer, which encircles the DNA and interacts with the DNA polymerase to bring them in close proximity to increase its processivity (Moldovan et al., 2007). A recent study showed that siRNA mediated depletion of RFC ATPase compromised the interaction between RFC and LANA and directly affected LANA-mediated replication and episome maintenance (Moldovan et al., 2007). Similarly, it has been shown that cellular mitotic checkpoint kinase, Bub1 plays a significant role in LANA mediated recruitment of PCNA to the TR to initiate viral replication in S phase (Sun et al., 2015). Bub1 establishes a molecular complex with PCNA and LANA and was found to be critical to promote LANA mediated mono-ubiquitination of PCNA (Sun et al., 2015). Two more cellular proteins shown to be critical for KSHV genome replication and maintenance are the components of Timeless-dependent DNA replication fork protection complex, Tim and Tipin (Dheekollu et al., 2013). These components are recruited to the TR in a LANA-dependent manner, suggesting their role in recombination-like structures formed during latent DNA replication (Dheekollu et al., 2013). Our studies have shown that LANA CBD specifically recruits cellular Topoisomerase II (TopoIIβ) to the TR region (Purushothaman et al., 2012). Topoisomerase II (TopoIIβ) is known to regulate and modify the DNA topology by introducing double-stranded breaks in DNA (Purushothaman et al., 2012). LANA localizes with the TopoIIβ in the nuclei of KSHV infected cells. Additionally LANA mediated recruitment of TopoIIβ to TR was also found to be critical for KSHV DNA replication in KSHV infected cells as well as transient replication system. Furthermore, KSHV infected cells treated with ellipticine, a TopoIIβ inhibitor, significantly reduced TR mediated replication (Purushothaman et al., 2012). These studies clearly indicate that LANA recruits multiple components of the cellular replication machinery at the origins within the TR to initiate latent DNA replication.

### KSHV ori-A

Accumulating evidence suggest that, KSHV latent DNA replication also can be initiated at multiple origins, other than ori-P (Verma et al., 2007b, 2011). Analysis of KSHV genome using Single Molecule Analysis of Replicated DNA (Verma et al., 2011; Adam et al., 2015), we identified an additional replication origin (ori-A), which did not require LANA for replication (Verma et al., 2011). SMARD is an ingenious method to accurately detect the origin and direction of fork progression on the replicating DNA, metabolically labeled with nucleoside analogs IdU and CldU (Norio and Schidkraut, 2001; Verma et al., 2011). SMARD analysis on the whole KSHV genome, during latent replication, determined that multiple sites in the genome could initiate replication rather than a specified region (Verma et al., 2011). Additionally, ChIP assay for ORC2 and MCM3 further confirmed the recruitment of these pre-replication complexes (pre-RC) at multiple genomic sites, confirming the formation of functional origins at those sites (Verma et al., 2011). However, these experiments consistently showed slight preference for replication initiation within the TR region. This data suggests that TR region of the KSHV genome have additional role in KSHV biology along with replicating the genome in a LANA dependent manner (Verma et al., 2011). Thus, KSHV latent DNA replication utilizes the cellular machinery to pursue a replication mechanism similar to the host DNA to maintain a life-long persistence (Verma et al., 2011).

## KSHV genome segregation

KSHV genomes replicate once per cell cycle in latent cells and are perpetually segregated into daughter cells along with host chromosomes during cell division (Ballestas et al., 1999; Barbera et al., 2006; Ueda et al., 2006). It has been shown that LANA associates with mitotic chromatin and plays a significant role in the maintenance of viral episomes during persistent infection (Ballestas et al., 1999; Cotter and Robertson, 1999; Tetsuka et al., 2004). The episomal tethering and segregation is effectively regulated by the specific association of LANA with histones (H2A/H2B) (Barbera et al., 2006; Verma et al., 2013) and a methyl CpG-binding protein (MeCP2) (Griffiths and Whitehouse, 2007; Matsumura et al., 2010; Verma et al., 2013) through its N-terminal chromosome binding region (CBD). Histones are small, conserved basic proteins that are involved in the organization of nucleosomes, the basic repeating units of condensed chromatin. Each core unit comprises two copies of histone H2A, H2B, H3, and H4 with 147 base pairs of DNA wrapped around this core particle. The nucleosomes are linked together by histone H1, which is also called the linker histone (Campos and Reinberg, 2009). It has been shown that LANA N terminus (LANA 1-32) directly interacts with histones H2A and H2B (H2A/B) (Luger et al., 1997; Cotter and Robertson, 1999). A recent study, further found that H2AX, an isoform of H2A can also interact with N and C terminals of LANA and contributes toward the phosphorylation of H2AX thereby aiding in binding of LANA at the TRs (Jha et al., 2013). Similarly, both LANA-N and LANA-C termini also were found to interact with MeCp2 and this association is suggested to favor the maintenance and segregation of KSHV genome (Krithivas et al., 2002). Methyl CpG binding protein 2 (MeCP2) is a nuclear protein, which specifically binds to methylated CpG dinucleotides and plays a key role in the transcriptional silencing of genes in CpG-methylated regions, activation of euchromatic genes, and mRNA splicing (Meehan et al., 1992; Nan et al., 1997; Aravind and Landsman, 1998). MeCP2 was also found to enhance the LANA-mediated activation of E2F1 promoter (Matsumura et al., 2010). Furthermore, LANA also interact with yet another nuclear protein DEK through the C-terminal DNA binding region (DBD), which is suggested to play a crucial role in KSHV genome tethering (Alexiadis et al., 2000; Krithivas et al., 2002). DEK has been shown to specifically interact with histones H2A, H2B, H3, and H4 during G1/S phase (Alexiadis et al., 2000; Waldmann et al., 2004). Direct interaction of LANA with DEK and DBD of LANA being the specific DEK binding domain, seemed to be crucial for KSHV genome tethering and segregation (Krithivas et al., 2002).

Additionally, LANA has been shown to interact with various nuclear matrix proteins required for the maintenance of nuclear architecture during mitosis, such as nuclear mitotic apparatus protein (NuMA), centromeric protein F (CENP-F) and kinetochore protein, Bub1 (Si et al., 2008; Xiao et al., 2010; Verma et al., 2013). NuMA is a nuclear-coiled coil protein, which is critical for organization of spindle apparatus and segregation of dividing nuclei during cell division (Guse et al., 2011). NuMA also plays a central role in DNA replication and transcription through its interaction with matrix attachment regions as well as the splicing factors, snRNPs (Doxsey et al., 2005). NuMa has been shown to be essential for persistence of KSHV genomes and their distribution to the daughter cells through its interaction with LANA at its C terminus and this association also requires the presence of dynein/dynactin and microtubules (Si et al., 2008). During interphase NuMA interacts with LANA through its C-terminus and plays a crucial role in the maintenance of nuclear architecture for episome segregation (Si et al., 2008). Immediately after the disintegration of nuclear envelope NuMA is hyper phosphorylated and moved to the spindle poles to secure the spindle microtubules to each pole (Merdes et al., 1996; Gehmlich et al., 2004). Importantly, NuMA knockdown has been shown to severely affect episome segregation as well as TR mediated genome maintenance (Si et al., 2008). Similarly, it has been shown that kinetochore proteins, Bub1 and CENP-F interact with the centromere and serve as the docking site of the spindle microtubules (Guse et al., 2011). Since centromeres are absent on viral genomes, association of KSHV LANA with kinetochore proteins, Bub1 and CENP-F are crucial for the segregation of episomes during cell division (Xiao et al., 2010). Both the N-terminal and C-terminal domains of LANA have been shown to interact with Bub1 and CENP-F (Xiao et al., 2010). These data strongly suggest that several proteins with in the nuclear matrix play significant role during KSHV DNA replication as well as genome segregation during mitosis (Xiao et al., 2010).

## Summary

Kaposi's sarcoma-associated herpesvirus (KSHV) persists predominantly as a latent episome in the infected cells. The latency-associated nuclear antigen (LANA) encoded by KSHV plays a central role in episome tethering, replication, and segregation of episomes during cell division. KSHV genome replicates once per cell cycle during latent replication and depends fully on cellular replication machinery for replication and genome maintenance. KSHV genome consists of LANA dependent replication origin, ori-P, within the TR region as well as additional replication sites distributed throughout the genome. ori-P consists of two LANA-binding sites (LBS) LBS-1/2 and a 32-bp GC-rich segment (32GC). KSHV encoded LANA is indispensable during latency and plays a crucial role in the replication mediated by ori-P. LANA binding to LBS sequence specifically recruit cellular replication factors to viral replication origin (ori-P) to initiate replication. KSHV recruits cellular pre-replication complexes (pre-RC) components such as origin recognition complexes (ORCs), cell division cycle (Cdc6) proteins and minichromosome maintenance proteins (MCM) to the ori-P. Additionally, LANA interacts with various nuclear matrix proteins required for the maintenance of nuclear architecture during mitosis, such as nuclear mitotic apparatus protein (NuMA), centromeric protein F (CENP-F) and kinetochore protein (Bub1). These specific interactions proved to be critical for the effective segregation of episomes into daughter cells. Taken together, the currently available data strongly suggests that KSHV LANA recruits several cellular proteins within the nuclear matrix to facilitate KSHV latent DNA replication as well as genome segregation during cell division.

## Author contributions

All authors listed, have made substantial, direct and intellectual contribution to the work, and approved it for publication.

### Conflict of interest statement

The authors declare that the research was conducted in the absence of any commercial or financial relationships that could be construed as a potential conflict of interest.
